# The Effects of Shock Wave Therapy on Spasticity and Walking Ability in People with Stroke: A Comparative Study of Different Application Sites

**DOI:** 10.3390/brainsci13040687

**Published:** 2023-04-20

**Authors:** Jung-Ho Lee, Eun-Ja Kim

**Affiliations:** Department of Physical Therapy, Kyungdong University, 815, Gyeonhwon-ro, Munmak-eup, Wonju-si 26495, Gang-won-do, Republic of Korea; ljhcivapt@naver.com

**Keywords:** neurorehabilitation, spasticity, gait ability, ischemic stroke

## Abstract

Background: This study was conducted to investigate the effects of extracorporeal shock wave therapy on the improvement of walking ability through a reduction in spasticity in stroke patients. Methods: Thirty-three patients diagnosed with ischemic stroke by a rehabilitation medicine specialist were randomly assigned to three groups. The patients were divided into experimental group 1 in which shock waves were applied to the muscle–tendon junction, experimental group 2 in which shock waves were applied to the middle of the muscle, and experimental group 3 in which shock waves were applied to both the muscle–tendon junction and the middle of the muscle. The MAS was used to evaluate spasticity in the subjects, and the Dartfish software was used to measure knee and ankle angles during heel-off when walking. Results: Based on the results of the study, a significant decrease in spasticity and increased joint angles were found in experimental groups 1 and 3 compared to experimental group 2, and the change in joint angle was significantly greater in experimental group 3 than in experimental groups 1 and 2. Conclusions: These results indicate that treatment effect may vary depending on the application site of the shock wave, and to obtain the best treatment effect, the shock wave should be applied to both the muscle–tendon junction and the middle part of the muscle.

## 1. Introduction

Stroke is a cerebrovascular disease caused by a disruption in blood flow to the brain. The two main types of strokes are ischemic and hemorrhagic strokes [1]. Ischemic strokes occur when a blood clot blocks a blood vessel in the brain, while hemorrhagic strokes result from bleeding in the brain due to a ruptured blood vessel. Regardless of the type of stroke, the resulting damage to the brain tissue can lead to a wide range of physical and cognitive impairments. The specific symptoms depend on the location and severity of the stroke, but they can include muscle weakness or paralysis, difficulty speaking or understanding language, vision problems, and memory loss [2].

The pathophysiology of stroke involves a complex series of events. In ischemic strokes, the blood clot blocks the blood vessel, preventing oxygen and nutrients from reaching the affected area of the brain. This leads to the death of brain cells in the affected region, which can cause lasting damage [3]. In hemorrhagic strokes, bleeding in the brain can cause increased pressure and swelling, which can also lead to brain cell death. Additionally, the presence of blood in the brain can trigger an inflammatory response that can further damage surrounding tissues [4].

The body’s response to stroke also plays a role in the overall damage and recovery process. Immediately following a stroke, the brain may go into a state of “neuronal shock”, during which the affected neurons are temporarily non-functional. Over time, the brain can initiate processes to repair and reorganize neural connections, although the extent and success of this recovery varies from patient to patient [5].

Stroke is a leading cause of movement disorders that can significantly affect a patient’s quality of life. Motor deficits can result from damage to the areas of the brain responsible for movement control and may result in muscle weakness, stiffness, or loss of coordination. Stiffness or increased muscle tone may also occur after a stroke, which makes movement more difficult and can contribute to muscle stiffness and pain. Spasticity can also interfere with a person’s ability to perform daily activities, such as reaching for and grasping objects [6]. In addition, coordination deficits, such as ataxia, can develop after a stroke, which can lead to difficulty with balance and coordination, making it difficult to walk or perform more complex movements [7].

Motor deficits following a stroke can have a significant impact on a patient’s ability to walk, also known as gait. Gait disturbances are common following a stroke and can have a significant impact on a patient’s functional independence and quality of life [8]. Hemiplegic gait, characterized by weakness or paralysis on one side of the body, is a common gait disturbance following a stroke. This can result in a slower walking speed, decreased step length on the affected side, and decreased overall walking distance. Patients may also exhibit compensatory movements, such as hip hiking or circumduction, in an attempt to compensate for the affected limb [9].

Spasticity can also contribute to gait disturbances following a stroke [10]. This can result in increased muscle tone, leading to stiffness and difficulty with movement. Patients may exhibit a “scissoring” gait pattern, characterized by crossing of the legs during walking, or toe walking due to increased tone in the calf muscles. The potential mechanisms of spasticity include changes in the properties of muscle fibers and their connections to the nervous system, as well as changes in the mechanical properties of the muscle–tendon unit. Spasticity can also be influenced by changes in the excitability of the spinal reflex pathways and the descending pathways from the brain [6,8]. Additionally, there may be alterations in the sensory feedback from the muscles and joints, which can contribute to the development of spasticity. In terms of expression patterns, spasticity can manifest as an increase in muscle tone, stiffness, and resistance to passive movement. This can lead to abnormal postures and movements, such as hyperextension or hyperflexion of joints, clenched fists, and curled toes [9,10]. Coordination deficits can also impact gait following a stroke. Ataxic gait, characterized by unsteadiness and a lack of coordination, can result in difficulty maintaining balance while walking. This can increase the risk of falls and further impair a patient’s mobility and independence [7].

Overall, motor deficits following a stroke can have a significant impact on a patient’s gait and walking ability. Effective rehabilitation strategies, such as gait training and targeted exercises to improve strength and coordination, are essential for improving gait and functional mobility in stroke patients [11].

Extracorporeal shock wave therapy (ESWT) has shown potential in reducing spasticity during walking through nerve regeneration in stroke patients [12]. ESWT is known to be effective in recovery of the damaged area by controlling the microenvironment through destruction of damaged tissues and cells, as well as promoting neovascularization and increasing growth factors by precisely exposing the affected area to a shock wave. Consequently, ESWT has been considered a therapeutic tool for stroke patients with dystonia, decubitus, or lymphedema, as well as patients with musculoskeletal disorders [13,14].

The focus type of extracorporeal shock wave therapy is a specific type of ESWT that utilizes a focused shock wave to deliver a more precise and targeted treatment to the affected area. This type of ESWT is also known as focused extracorporeal shock wave therapy or simply shock wave therapy. Unlike radial shock wave therapy, which uses a radial or dispersed shock wave to treat a broader area, the focus type of ESWT concentrates the shock wave to a specific focal point. This focal point is determined by the size and shape of the applicator used, which can vary in size depending on the area being treated [15].

The focused shock wave can penetrate deeper into the tissue and produce a higher level of energy density at the focal point. This increased energy density can result in a more intense and targeted therapeutic effect. The focus type of ESWT is commonly used to treat conditions that are difficult to heal, such as tendinopathies, plantar fasciitis, and bone fractures [16].

ESWT is a non-invasive medical procedure that utilizes low-intensity acoustic waves to treat various musculoskeletal conditions. Although the exact mechanism of ESWT is not yet fully understood, it is believed to involve multiple factors that contribute to its therapeutic effect [12]. One of the most significant factors in ESWT’s mechanism is the stimulation of growth factors. Shockwaves are believed to stimulate the release of growth factors, such as vascular endothelial growth factor, which promotes angiogenesis and tissue regeneration. By increasing blood flow and the formation of new blood vessels, ESWT can help repair damaged tissues and improve their function [17].

ESWT may also have a mechanical effect on the affected tissue. Its shock waves can disrupt calcifications and fibrosis in the affected tissue. This is thought to occur through a mechanical effect, which can break down calcified deposits and loosen fibrotic tissue. This mechanism can be particularly useful for conditions such as plantar fasciitis or tendinopathies [18]. Furthermore, ESWT may have an analgesic effect by stimulating the release of endorphins and reducing pain signals in the affected area. This can help alleviate pain associated with musculoskeletal conditions and promote healing [19]. Another potential mechanism of ESWT is its anti-inflammatory effect. Shock waves can reduce the expression of pro-inflammatory cytokines and promote the release of anti-inflammatory cytokines, leading to a reduction in inflammation in the affected area. This mechanism can be especially beneficial for conditions such as osteoarthritis or tendinitis [20].

In summary, ESWT’s therapeutic effect is believed to be due to a combination of factors, including the stimulation of growth factors [17], the mechanical disruption of calcifications and fibrosis [18], the analgesic effect [19], and the anti-inflammatory effect [20]. However, further research is needed to fully understand the mechanism of ESWT and to optimize its clinical use.

There is a lack of research on the use of extracorporeal shock wave therapy for improving gait in stroke patients through the reduction in spasticity. While ESWT has been studied for its potential to reduce spasticity in other patient populations, such as those with cerebral palsy, the research on its use in stroke patients is limited [21]. Some studies have suggested that ESWT may be effective for reducing spasticity in stroke patients, leading to improved functional outcomes, including gait [12]. However, the existing research is limited in terms of sample size and study design, and more rigorous studies are needed to determine the effectiveness of ESWT for improving gait in stroke patients through the reduction in spasticity. Therefore, in this study, extracorporeal shock wave therapy was applied to reduce spasticity and improve walking ability in stroke patients. Through this, the clinical effect of shock wave therapy was evaluated, and the optimal application site was determined.

## 2. Materials and Methods

### 2.1. Subjects

This study selected 33 subjects diagnosed with ischemic stroke by a specialist in rehabilitation medicine. The subjects were randomly assigned to three groups: experimental group 1, which received shock waves applied to the muscle–tendon junction; experimental group 2, which received shock waves applied to the mid-belly of the muscle; and experimental group 3, which received shock waves applied to both the muscle–tendon junction and the mid-belly of the muscle. Before the experiment, the subjects were asked to choose a card labeled A, B, or C, and those who chose A were assigned to experimental group 1, those who chose B were assigned to experimental group 2, and those who chose C were assigned to experimental group 3. The subjects in all experimental groups received extracorporeal shock wave therapy (ESWT) after undergoing a general physical therapy session, which included 30 min of upper- and lower-extremity manual therapy based on proprioceptive neuromuscular facilitation.

**Figure 1:**
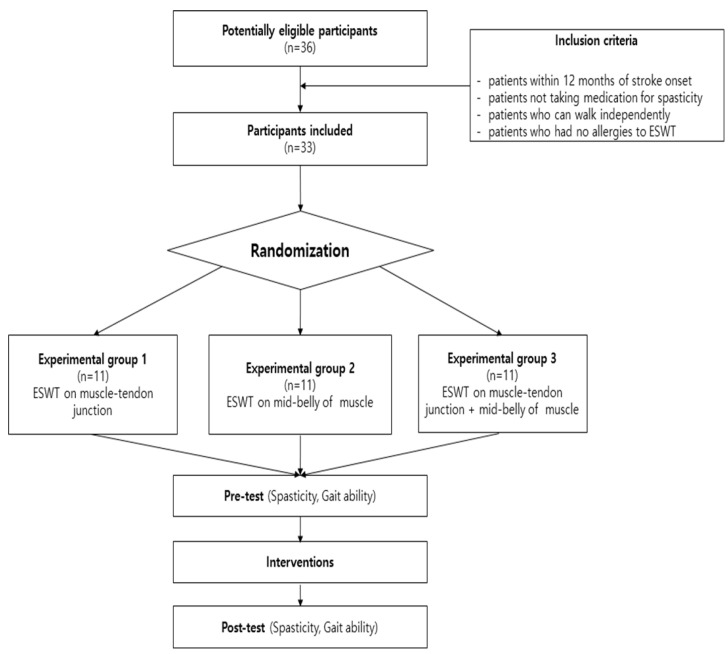
Flowchart of research.

The selection criteria for the subjects in this study included patients with an ischemic stroke occurring within 12 months, patients who were capable of independent walking, patients who were not receiving medication for reducing spasticity, and patients who had no allergies to shock wave therapy (Figure 1). This study was conducted with patients with a Mini-Mental State Examination (MMSE) score of 24 or higher. This study obtained the calculation values for the sample size, power, and effect sizes using the G*Power software version 3.1(HHU, Düsseldorf, Germany). The sample size was determined based on a power analysis with an alpha level of 0.05 and a power of 0.80. The effect size was estimated to be 0.5, which is considered a moderate effect size. Based on these parameters, a sample size of 33 patients was determined to be appropriate for this study.

In accordance with the Helsinki Declaration for patient participation in research, prior to the clinical trial, all patients were provided with an explanation of the study’s overall purpose, process, risks, and potential side effects. They were also given the opportunity to ask questions and seek clarification before voluntarily consenting to participate in the study. The research procedures were conducted under the supervision of the local Institutional Review Board, which ensures the ethical and legal conduct of research, and all experimental procedures and protocols were approved by the research ethics committee of the relevant university.

### 2.2. Intervention Methods

A focused extracorporeal shock wave therapy device (Optimus, Salus Talent 3, Seongnam-si, Korea) was used to apply shock waves to the triceps surae muscle in this study. The shock waves applied to the experimental groups 1 and 2 had a frequency of 2 Hz, an energy density of 0.1 mJ/mm^2^, and a total of 6000 pulses per treatment session. The therapy was administered once a day, five times a week, for a total of 20 sessions over four weeks. In the experimental group 3, shock waves with the same frequency and energy density were applied, but 3000 pulses were applied to the muscle–tendon junction and 3000 pulses were applied to the mid-belly of the muscle, for a total of 6000 pulses per treatment session. The therapy was also administered once a day, five times a week, for a total of 20 sessions over four weeks.

### 2.3. Assessment Methods

For all evaluations, a pre-test was performed before the first intervention, and a post-test was carried out after the last intervention at the end of four weeks.

#### 2.3.1. Method for Assessment of Spasticity of Lower Extremities

In this study, the Modified Ashworth Scale (MAS) was used to evaluate spasticity in stroke patients. The Modified Ashworth Scale is a widely used clinical tool for assessing spasticity in patients with neurological disorders, including stroke, and the MAS assesses spasticity based on resistance felt during passive movement. After passively moving a patient’s limbs through the full range of motion, the physical therapist performing the evaluation in this study evaluated the level of resistance felt during movement on a 6-point scale ranging from 0 to 4. In general, higher scores on the MAS indicate greater spasticity and muscle tone, with a score of 0 indicating normal muscle tone.

Scores range from 0 for no increase in muscle tone to 4 for affected part(s) that are rigid in flexion or extension. Scores of 1 and 1+ indicate a slight increase in muscle tone, while a score of 2 indicates a more marked increase in muscle tone. Scores of 3 and 4 indicate considerable increase in muscle tone and passive movement difficulties, respectively.

For statistical analysis in this study, a score of 1+ was considered as 1.5 points. All evaluations were performed by one physical therapist with more than 10 years of clinical experience, and the evaluator was blinded to which experimental group the participants belonged to. In addition, a pre-evaluation was conducted before the application of the shock wave therapy, and a post-evaluation was performed after the final therapy session.

#### 2.3.2. Assessment of Gait

In this study, the Dartfish (myDartfish Express PC version 10.0, DFKOREA, Hanam-si, Korea) software was used to evaluate the patients’ gait ability. Dartfish is a software used for gait analysis, which involves a person’s walking pattern. This program uses motion analysis to track a person’s movement during walking and can be used to assess the person’s gait mechanics, such as joint angles, step length, and stride length.

In this study, to measure and compare changes in knee and ankle angles during the heel-off phase when walking, a camera was fixed outside at a distance of 3 m while the participants walked 10 m on a flat surface. Reflective markers were attached to the anatomical locations of the greater trochanter and lateral epicondyle of the femur, the lateral malleolus of the tibia, and the head of the fifth toe to measure the angles of the knee and ankle joints.

The angles of the knee and the ankle joints’ internal angles were measured using the Dartfish software by selecting only videos when the participants performed complete steps. The angles were measured at the point where the extension lines connecting each marker met during walking. After a total of three evaluations per subject, the average value was used as the measured value.

### 2.4. Data Analysis

The data collected to evaluate the patients’ general characteristics, lower extremity muscle stiffness, and medial angle of the knee and ankle joints were analyzed using SPSS 18.0 (SPSS inc. Chicago, IL, USA)for Windows. Normality was verified using the Shapiro–Wilk test, and all data were described as mean ± standard deviation using descriptive statistics.

A one-way analysis of variance was used to test the homogeneity of the general characteristics and pre-assessment of the subjects, and a paired t-test was conducted to find differences between the pre-test and post-test within the A, B, and C groups. One-way analysis of variance was performed using the amount of change between the pre-test and post-test as the dependent variables to determine differences in treatment effects between groups, with Tukey’s HSD as a post hoc test. The significance level was set at α < 0.05.

## 3. Results

### 3.1. General Characteristics of Subjects and Homogeneity Test of Pre-Test Measurements

The study subjects’ general characteristics and pre-test measurements of the dependent variables, including MAS, knee angle, and ankle angle, were homogenous with no statistically significant differences, as shown in Table 1.

### 3.2. Changes in Spasticity

This study investigated the effect of extracorporeal shock wave therapy on reducing muscle spasticity according to the application area. In group EX 1, the pre-test and post-test scores for knee extensor (KE) were 2.63 ± 0.63 and 1.95 ± 0.41, respectively, with a statistically significant reduction in spasticity (*p* = 0.002). The t-value was 4.038, indicating a moderate effect size. In group EX 2, the pre-test and post-test scores for KE were 2.59 ± 0.58 and 1.86 ± 0.45, respectively, with no significant reduction in spasticity (*p* = 0.082). The t-value was 1.936, indicating a small effect size. In group EX 3, the pre-test and post-test scores for KE were 2.68 ± 0.56 and 1.86 ± 0.23, respectively, with a statistically significant reduction in spasticity (*p* = 0.000). The t-value was 8.050, indicating a large effect size (Table 2).

In the evaluation of ankle flexor (AF) muscle spasticity in this study, group EX 1 demonstrated a significant reduction in spasticity with a large effect size (t = −6.333, *p* = 0.000), as indicated by the pre- and post-test scores of 2.72 ± 0.46 and 1.86 ± 0.45, respectively. Group EX 2 showed no significant reduction in spasticity of AF muscles with a small effect size (t = −1.838, *p* = 0.096), as evidenced by the pre- and post-test scores of 2.95 ± 0.56 and 2.72 ± 0.64, respectively. In contrast, group EX 3 showed a significant reduction in spasticity of AF muscles with a large effect size (t = −6.901, *p* = 0.000), as shown by the pre- and post-test scores of 2.81 ± 0.40 and 1.90 ± 0.43, respectively.

Overall, the results show that ESWT on the muscle–tendon junction location can significantly reduce muscle spasticity, whereas ESWT on the mid-belly location may not be effective. However, the effectiveness of ESWT may vary depending on the treatment location, as seen in the significant reduction in spasticity when ESWT was performed on both the muscle–tendon junction and mid-belly locations in group EX 3.

One-way analysis of variance was performed using the amount of change between the pre-test and post-test as the dependent variables to determine differences in treatment effects between groups, and the results are presented in Table 3. In the comparison between the groups for knee extensors and knee flexors, experimental group 1 and experimental group 3 showed a statistically significant difference in treatment effect compared to experimental group 2. However, there was no significant difference between experimental group 1 and experimental group 3.

### 3.3. Changes in Gait Ability

Table 4 shows the comparison of gait ability during heel-off between the three groups of stroke patients. The knee joint angle (KA) and ankle joint angle (AA) were measured during the pre-test and post-test. In group EX 1, the pre-test and post-test knee joint angles were 20.45 ± 2.87 and 18.72 ± 1.84 degrees, respectively, with a statistically significant reduction (*p* = 0.007). The t-value was 3.413, indicating a moderate effect size. In group EX 2, the pre-test and post-test knee joint angles were 20.27 ± 2.45 and 19.90 ± 2.25 degrees, respectively, with a statistically significant increase (*p* = 0.221) in the joint range of motion. The t-value was 1.305, indicating a small effect size. In group EX 3, the pre-test and post-test knee joint angles were 18.81 ± 1.66 and 16.54 ± 1.63 degrees, respectively, with a statistically significant reduction (*p* = 0.000). The t-value was 6.829, indicating a large effect size.

In the evaluation of ankle joint angle in this study, group EX 1 demonstrated a significant reduction in spasticity with a large effect size (t = −6.633, *p* = 0.000), as indicated by the pre- and post-test scores of 11.45 ± 1.50 and 13.45 ± 1.12, respectively. Group EX 2 showed no significant increase in the range of motion for ankle joint with a small effect size (t = −1.472, *p* = 0.172), as evidenced by the pre- and post-test scores of 11.81 ± 2.04 and 12.45 ± 2.11, respectively. In contrast, group EX 3 showed a significant increase in the range of motion for ankle joint with a large effect size (t = −9.023, *p* = 0.000), as shown by the pre- and post-test scores of 12.90 ± 1.57 and 16.00 ± 1.18, respectively.

In this study, the differences in treatment effects between groups according to the changes in knee and ankle angles were investigated using one-way ANOVA (Table 5). One-way analysis of variance was performed using the amount of change between the pre-test and post-test as the dependent variables to determine the differences in treatment effects between groups. The study results showed that there were significant differences in knee and ankle angle changes between the three experimental groups, with experimental group 1 and experimental group 3 demonstrating greater changes than experimental group 2. Furthermore, experimental group 3 showed significantly greater angle changes of ankle joint compared to experimental group 1.

The study results suggest that applying extracorporeal shock wave therapy at specific locations can effectively increase the range of motion in stroke patients. The increasing range of motion was evaluated by analyzing knee and ankle joint angles during gait, and significant improvements were observed in the experimental groups that received ESWT at the muscle–tendon junction and mid-belly of the muscle compared to the group that received ESWT at the mid-belly of the muscle only. Therefore, the location of ESWT application is a crucial factor in determining its effectiveness in increasing range of motion in stroke patients. These findings could have important implications for the development of future treatment strategies for stroke patients.

## 4. Discussion

After a central nervous system (CNS) injury, such as stroke, traumatic brain injury, or spinal cord injury, the neural pathways that control muscle tone and movement can be disrupted. This disruption can lead to an imbalance of excitatory and inhibitory signals from the brain to the muscles, resulting in increased muscle tone and stiffness known as spasticity [22].

The exact mechanism underlying spasticity is not fully understood, but it is believed to involve changes in the properties of neurons and their connections in the CNS. After an injury, there is an increase in the release of excitatory neurotransmitters, such as glutamate, and a decrease in the release of inhibitory neurotransmitters, such as gamma-aminobutyric acid. This results in an overall increase in neural excitability, leading to hyperactive reflexes and spasticity. Additionally, there may be changes in the sensitivity of muscle fibers and their connections to the nervous system. These changes can result in a state of constant contraction known as hypertonicity, which contributes to spasticity [23,24].

Increased spasticity after a stroke can have a significant impact on a patient’s upper and lower limbs and ability to maintain balance. Spasticity can cause muscle stiffness and rigidity, which can limit joint movement and impair the ability to perform daily activities. This can lead to difficulties in walking, standing, and maintaining balance, which can increase the risk of falls and injuries [10,12].

In the upper limbs, spasticity can cause muscle contractions, which can result in abnormal positioning of the arms and hands. This can affect a patient’s ability to perform fine motor tasks, such as writing, grasping objects, and dressing themselves. It can also cause pain, discomfort, and muscle weakness, which can further impair their ability to use their upper limbs [25]. In the lower limbs, spasticity can cause muscle stiffness and tightness, which can affect a patient’s ability to walk and maintain balance. It can cause abnormal gait patterns, such as foot drag, toe walking, and scissoring, which can increase the risk of falls and injuries. Spasticity can also cause muscle fatigue, which can lead to decreased endurance and mobility [26].

Gait disturbances are a common issue for stroke patients, and poor knee and ankle angles have been associated with these impairments [8]. In stroke patients with gait disturbances, a reduced range of motion in the ankle joint can result in foot clearance issues during the swing phase of gait. This can cause a “drop foot” gait pattern, where the toes scrape the ground with each step [27]. A reduced range of motion in the knee joint can also cause problems, such as a crouched gait pattern with excessive knee flexion during the stance phase. On the other hand, hyperextension of the knee can lead to a “back-knee” gait pattern, where the knee moves backward during the stance phase. This can cause instability during walking and increase the risk of falls [28].

There are a variety of clinical treatment modalities used to reduce spasticity in stroke patients. One common treatment is physical therapy, which includes a range of exercises and techniques to help improve muscle strength and flexibility and promote motor function [29]. Physical therapy can be tailored to an individual patient’s needs and can include stretching exercises, range-of-motion exercises, and strengthening exercises. Additionally, there are specialized physical therapy techniques, such as neurodevelopmental treatment and constraint-induced movement therapy, that may be utilized [30].

Other treatment modalities include medications, such as muscle relaxants and antispasmodics, which can help reduce spasticity in stroke patients by targeting the central nervous system and reducing the activity of neurons that cause spasticity [31]. Botulinum toxin injections may also be used to temporarily paralyze muscles and reduce spasticity [32]. Electrical stimulation is another treatment modality that involves applying an electrical current to the affected muscle. This can help reduce stiffness and improve muscle function [33]. In addition, orthoses, such as braces and splints, may be used to support weakened or spastic muscles and promote better alignment [34].

While these treatments can be effective in reducing spasticity in stroke patients, they also have potential disadvantages, such as side effects from medications and the cost and inconvenience of repeated injections. Furthermore, some patients may not respond well to certain treatments, and there is no single treatment that works for everyone [35].

The selection of an appropriate treatment method that is suitable for a patient’s specific condition is an essential aspect of successful rehabilitation. By choosing a treatment approach that has fewer side effects and is more tolerable for the patient, it is more likely that the patient will continue to attend treatment and ultimately achieve better outcomes. In the case of spasticity reduction in stroke patients, ESWT has shown promising results with minimal side effects, making it a potentially valuable addition to the existing repertoire of treatment modalities [36].

The exact mechanism by which ESWT reduces spasticity in stroke patients is not yet fully understood, but there are several proposed mechanisms. One proposed mechanism is that ESWT disrupts the abnormal muscle tone by decreasing the excitability of the stretch reflex loop. The shock waves may activate afferent nerve fibers, which in turn inhibit alpha motor neurons, leading to reduced muscle tone. Additionally, ESWT may stimulate the release of growth factors, such as brain-derived neurotrophic factor, which can promote neuronal plasticity and lead to the rewiring of neural circuits involved in spasticity. Finally, ESWT may have a direct effect on muscle fibers, causing changes in the mechanical properties of the muscle and reducing spasticity [37].

Several studies have investigated the use of extracorporeal shock wave therapy to reduce spasticity in stroke patients. One study found that ESWT significantly reduced spasticity in the upper limbs of stroke patients. This study used a low-energy ESWT method and applied the therapy to the muscles and tendons around the affected joint [38]. Another study investigated the effects of ESWT on spasticity and motor function in stroke patients. This study used high-energy ESWT and applied the therapy to the ankle plantar flexor muscles. The results showed a significant reduction in spasticity and improvement in motor function in the experimental group [39]. The studies examined suggest that extracorporeal shock wave therapy could be a useful treatment choice for reducing spasticity in individuals who have suffered from a stroke.

In this study, the researchers focused on investigating the therapeutic effect of shock waves on spasticity reduction and determining the optimal method of shock wave application based on the theory and mechanism related to nerve regeneration. The results of the study showed that shock wave application to the muscle–tendon junction was more effective than shock wave application to the middle part of the muscle in reducing spasticity and increasing the range of motion of the joint, and this difference was statistically significant. These findings suggest that the location of shock wave application plays a critical role in the effectiveness of ESWT in reducing spasticity in stroke patients. In addition to the significant reduction in spasticity and improvement in the joint range of motion observed in experimental group 1 and experimental group 2, experimental group 3 showed even greater therapeutic effects. This suggests that the combined application of shock waves to both areas may have a synergistic effect on reducing spasticity and improving joint function. Additionally, it is believed that the positive effects observed are due to changes in the microenvironment, increased release of substances that promote nerve regeneration, and stimulation of sensory nerves resulting from the administration of ESWT. However, the limitations of this study include the non-randomized assignment of participants to the experimental groups, which might have introduced bias and impacted the generalizability of the findings. Secondly, although we estimated the effect size based on previous studies, it is possible that we might have overestimated the effect size. This could impact our sample size and statistical power, indicating that caution should be exercised when interpreting our study results. Additionally, the generalizability of our study findings may be limited, as the potential overestimation of the effect size could lead to differences between our results and those of other research or real-world clinical situations. Another constraint is the small sample size, which could limit the statistical power of the study. Furthermore, the precise mechanism behind the therapeutic effect of ESWT in reducing spasticity remains unclear, emphasizing the need for additional research to clarify the underlying biological mechanisms.

## 5. Conclusions

The findings of this paper indicate that extracorporeal shock wave therapy holds promise as a potential treatment for reducing spasticity in individuals who have suffered a stroke. The findings also suggest that the effectiveness of ESWT may be dependent on the specific site of application, with optimal results achieved when applying shock waves to both the muscle–tendon junction and mid-muscle region. This underscores the importance of tailoring ESWT to an individual patient’s characteristics, including the location and severity of spasticity and overall health status, in order to achieve the best possible outcomes. These results suggest that ESWT may be a valuable addition to a comprehensive rehabilitation program for stroke patients with spasticity.

## Figures and Tables

**Table 1:** General characteristics of subjects and homogeneity test of pre-test measurements.

	EX 1 (*n* = 11)	EX 2 (*n* = 11)	EX 3 (*n* = 11)	*p*
Age (years)	67.72 ± 4.42	68.09 ± 5.64	66.63 ± 5.12	0.786
Height (cm)	162.45 ± 9.88	159.72 ± 9.13	163.81 ± 8.90	0.583
Weight (kg)	58.09 ± 8.11	56.00 ± 8.37	57.54 ± 7.54	0.819
MMSE (score)	25.72 ± 1.55	25.63 ± 1.28	26.09 ± 1.22	0.712
MAS of knee extensor (score)	2.63 ± 0.63	2.59 ± 0.58	2.68 ± 0.56	0.938
MAS of ankle flexor (score)	2.72 ± 0.46	2.95 ± 0.56	2.81 ± 0.40	0.548
Knee angle (°)	20.45 ± 2.87	20.27 ± 2.45	18.81 ± 1.66	0.227
Ankle angle (°)	11.45 ± 1.50	11.81 ± 2.04	12.90 ± 1.57	0.138

Mean ± SD: mean ± standard deviation; MMSE: Mini-Mental State Examination; EX1: experimental group 1; EX2: experimental group 2; EX3: experimental group 3.

**Table 2:** Comparative analysis of changes in MAS.

Group	Muscles	Pre-Test	Post-Test	*t*	*p*
EX 1 (*n* = 11)	KE	2.63 ± 0.63	1.95 ± 0.41	4.038	0.002 *
	AF	2.72 ± 0.46	1.86 ± 0.45	6.333	0.000 *
EX 2 (*n* = 11)	KE	2.59 ± 0.58	2.31 ± 0.56	1.936	0.082
	AF	2.95 ± 0.56	2.72 ± 0.64	1.838	0.096
EX 3 (*n* = 11)	KE	2.68 ± 0.56	1.86 ± 0.23	8.050	0.000 *
	AF	2.81 ± 0.40	1.90 ± 0.43	6.901	0.000 *

Abbreviations: Number (*n*); EX1: ESWT on muscle–tendon junction; EX2: ESWT on mid-belly of the muscle; EX3: ESWT on muscle–tendon junction and mid-belly of the muscle; KE: knee extensor; AF: ankle flexor; *: paired *t*-test at *p* < 0.05; Unit: score. Note: Data are reported as mean ± SD.

**Table 3:** Comparative analysis between groups using the average value of change between pre-test and post-test for MAS.

	EX 1	EX 2	EX 3	*F*	*p*	Post Hoc
KE	0.68 ± 0.56	0.27 ± 0.46	0.81 ± 0.33	4.120	0.026 *	EX1, EX3 > Ex2
AF	0.86 ± 0.45	0.22 ± 0.41	0.90 ± 0.43	8.508	0.001 **	EX1, EX3 > Ex2

Mean ± SD: mean ± standard deviation; EX1: experimental group 1; EX2: experimental group 2; EX3: experimental group 3; KE: knee extensor; AF: ankle flexor; * *p* < 0.05; ** *p* < 0.01; Unit: score.

**Table 4:** Comparison of gait ability during heel-off between groups.

Group	Muscles	Pre-Test	Post-Test	*t*	*p*
EX 1 (*n* = 11)	KA	20.45 ± 2.87	18.72 ± 1.84	3.413	0.007 *
	AA	11.45 ± 1.50	13.45 ± 1.12	−6.633	0.000 *
EX 2 (*n* = 11)	KA	20.27 ± 2.45	19.90 ± 2.25	1.305	0.221
	AA	11.81 ± 2.04	12.45 ± 2.11	−1.472	0.172
EX 3 (*n* = 11)	KA	18.81 ± 1.66	16.54 ± 1.63	6.829	0.000 *
	AA	12.90 ± 1.57	16.00 ± 1.18	−9.023	0.000 *

Abbreviations: Number (*n*); EX1: ESWT on muscle–tendon junction; EX2: ESWT on mid-belly of the muscle; EX3: ESWT on muscle–tendon junction and mid-belly of the muscle; KA: knee joint angle; AA: ankle joint angle; *: paired *t*-test at *p* < 0.05; Unit: angle. Note: Data are reported as mean ± SD.

**Table 5:** Comparative analysis between groups using the average value of change between pre-test and post-test for gait ability.

	EX 1	EX 2	EX 3	*F*	*p*	Post Hoc
KA	1.72 ± 1.67	0.36 ± 0.92	2.27 ± 1.10	6.524	0.004 **	EX1, EX3 > Ex2
AA	−2.00 ± 1.00	−0.63 ± 1.43	−3.09 ± 1.13	11.485	0.000 **	EX1, EX3 > Ex2 EX3 > EX1

Mean ± SD: mean ± standard deviation; EX1: experimental group 1; EX2: experimental group 2; EX3: experimental group 3; KA: knee joint angle; AA: ankle joint angle; ** *p* < 0.01; Unit: angle.

## Data Availability

The datasets generated during the current study are available from the corresponding author upon reasonable request.
